# WONKA: objective novel complex analysis for ensembles of protein–ligand structures

**DOI:** 10.1007/s10822-015-9866-z

**Published:** 2015-09-19

**Authors:** A. R. Bradley, I. D. Wall, F. von Delft, D. V. S. Green, C. M. Deane, B. D. Marsden

**Affiliations:** SGC, Nuffield Department of Medicine, University of Oxford, Old Road Campus Research Building, Roosevelt Drive, Headington, Oxford, OX3 7DQ UK; Oxford Protein Informatics Group, Department of Statistics, University of Oxford, 1 South Parks Road, Oxford, OX1 TG UK; Computational & Structural Chemistry, GlaxoSmithKline, Medicines Research Centre, Gunnels Wood Road, Stevenage, Hertfordshire, SG1 2NY UK; Diamond Light Source Ltd, Harwell Science and Innovation Campus, Didcot, OX11 0QX UK; Department of Biochemistry, University of Johannesburg, Aukland Park, 2006 South Africa; Kennedy Institute of Rheumatology, Nuffield Department of Orthopaedics, Rheumatology and Musculoskeletal Sciences, University of Oxford, Roosevelt Drive, Headington, Oxford, OX3 7FY UK

**Keywords:** Structure based drug design, Data driven drug design, Bromodomains

## Abstract

WONKA is a tool for the systematic analysis of an ensemble of protein–ligand structures. It makes the identification of conserved and unusual features within such an ensemble straightforward. WONKA uses an intuitive workflow to process structural co-ordinates. Ligand and protein features are summarised and then presented within an interactive web application. WONKA’s power in consolidating and summarising large amounts of data is described through the analysis of three bromodomain datasets. Furthermore, and in contrast to many current methods, WONKA relates analysis to individual ligands, from which we find unusual and erroneous binding modes. Finally the use of WONKA as an annotation tool to share observations about structures is demonstrated. WONKA is freely available to download and install locally or can be used online at http://wonka.sgc.ox.ac.uk.

## Introduction

In recent years approaches to Structure Based Drug Design (SBDD) and specifically Fragment Based Drug Design [1–3] (FBDD) have developed extensively, resulting in a rapidly increasing number of available liganded structures for a given protein target. In the pharmaceutical industry it is now commonplace to have access to many tens of liganded crystal structures within a drug discovery programme, leading to the rise of a new field of Ensemble Based Drug Design [4], EBDD. A leading example of which being Pitt et al.’s Polyphony [4], which generates novel superimposition-dependent conformational analyses of large numbers (hundreds) of crystal structures. In general, however, computational tools have not kept pace with the surge in availability of this data and the workhorse for inspecting SBDD output remains generic structural biology visualisation tools such as Pymol [5]. However, such tools are designed toward the evaluation of at most a handful of structures at any one time. Furthermore they do not, by default, identify and highlight trends within a dataset that can be used to inform SBDD. Analysis methods that are available, e.g. pharmacophore model generation, produce generalised models that do not refer directly back to each ligand in an ensemble. Finally there is no approach allowing the user to easily capture and share observations of interest whilst inspecting structures. Any useful observations must be separately and manually added to online encyclopaedias such as Proteopedia [6]. As a result the inspection of SBDD output is currently a time consuming manual bottleneck, relying on the skill of the scientist to remember features between structures, thereby leading to repetitive, incomplete and subjective analyses.

Rather than acting as a bottleneck, the recent influx of data should present an opportunity to develop novel tools that present simple analyses of protein–ligand interactions to enable EBDD. In this work we focus on three core areas, ligand-based, residue-based and water-based analyses, since these are three of the most commonly considered structural biology analyses.

Ligand-based pharmacophore abstractions of structural data are commonly used in computational chemistry [7, 8], in large part by generating predictive three or four point pharmacophores from a single or a handful of ligands. These are useful for virtual screening exercises [9, 10], however for analyses of ensembles of liganded structures they present three major weaknesses. Firstly, they are by definition simplistic and reductionist models that cannot consider either the complete range of binding modes or infrequently represented features in the data. Secondly, the relative contribution of each ligand’s features to the pharmacophore model is not readily apparent resulting in analyses tending to be confined to broad trends, thereby potentially missing information about interesting compounds that do not fit general patterns. Thirdly, there is normally no connection between the ligand-based pharmacophore and its environment—interactions with protein and water atoms.

Further to ligand-based pharmacophores, presenting an overview of the effects of various ligands on residue conformation and water conservation/displacements are also key areas of interest when studying protein–ligand interactions [4, 11]. A number of tools exist to make predictions about these effects, but, to our knowledge, none exist to look for trends and extract important information from existing experimental data. Molecular dynamics and crystallographic methodologies [12] often cluster residue conformations to classify normal modes and residue interconnectivity [13]. Equally, energetics calculations can calculate the probability of waters being displaced [14, 15] upon ligand binding. Family-wide analyses can determine the likely spatial conservation of a given water ligand [16]. Each of these methods produces useful insights for SBDD, however they suffer from two key weaknesses. Firstly, they tend to be computationally expensive and complex, requiring careful configuration for each novel target class. Secondly, and most crucially, they are not designed with the goal of analysing ensembles of liganded structures. This means they are not able to directly refer their water-based and residue-based analyses of the ensemble back to the contributing ligands. It is the ability to not just identify patterns, but to easily relate these observations to individual ligand complexes that represents a gap in current computational capabilities.

To address these challenges we present WONKA, an automated computational tool that provides a range of analyses of protein–ligand structural ensembles. WONKA summarises ligands within binding site clusters, presents a pharmacophore-based analysis of core binding modes and summarises changes in residue conformation and water conservation across an ensemble of structures of the same protein. WONKA differs from currently available methods in three core ways. Firstly it presents combined ligand and protein based analyses in a single and intuitive web-based workflow. Secondly it relates these analyses at the level of individual ligands allowing for specific and nuanced interpretation. Finally the WONKA graphical user interface facilitates data annotation and sharing.

Below we describe the WONKA method for finding, consolidating and then visualising interesting features within an ensemble of structures. We then outline WONKA’s use in performing target-level comparisons between three Human bromodomain proteins. Finally we demonstrate WONKA’s ability to find interesting and erroneously modelled ligands within a dataset and then share these observations broadly.

## Method and materials

**Fig. 1:**
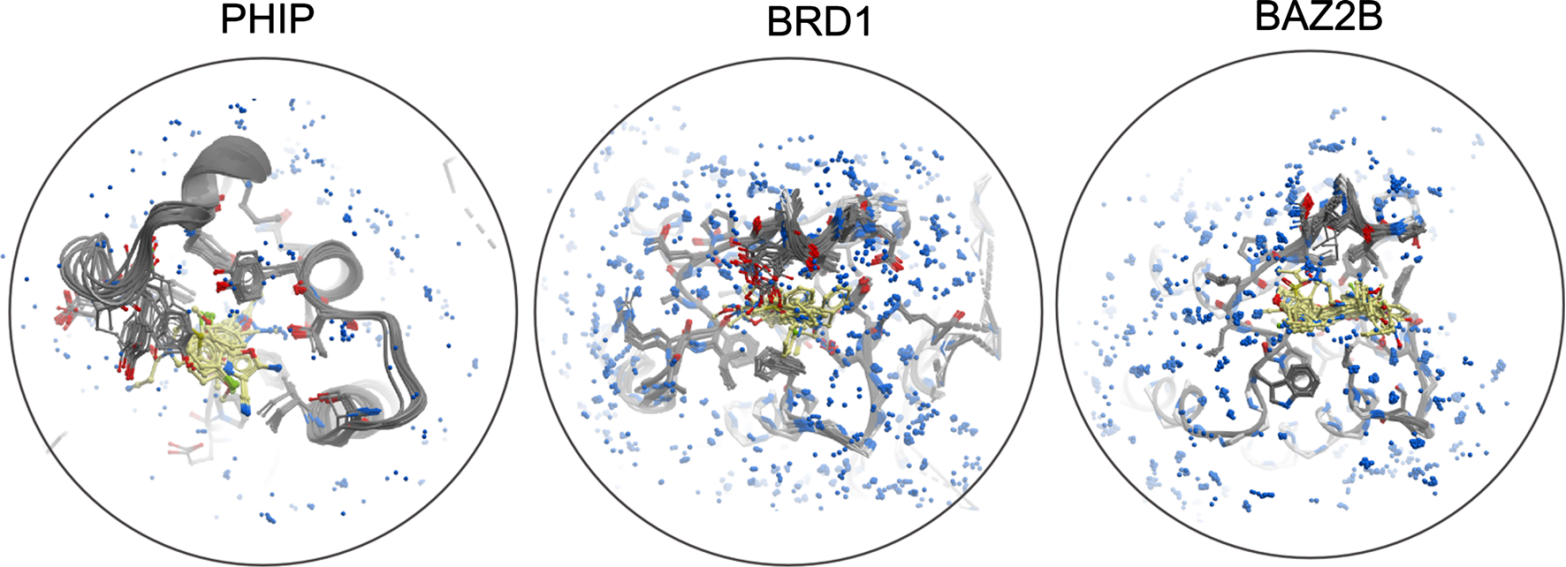
The ensemble of liganded structures for Human PHIP, BRD1 and BAZ2B bromodomains respectively (*left* to *right*). The superimposition of structures results in a visualisation which is extremely difficult to interpret, especially when attempting to identify nuanced changes presented in a minority of structures. Protein carbon in *grey* and ligand carbon in *yellow*. Heteroatoms are coloured according to Molsoft ICM colour scheme. *Blue* spheres are waters

In this paper we present the analyses of the following Human proteins: the second bromodomain of Human Pleckstrin homology domain interacting protein (PHIP—Uniprot Q8WWQ0), the bromodomain of Human bromodomain containing protein 1 (BRD1—Uniprot Q86X06) and the bromodomain of Human bromodomain adjacent to zinc finger domain 2B (BAZ2B—Uniprot Q9UIF8). The datasets consist of 13, 29 and 33 structures respectively. All are derived by X-ray crystallography and have a resolution better than 2.5 Å. All datasets were collected and analysed at the Diamond Light Source I04-1 beamline. Structures were superposed using Molsoft ICM’s [17] all residue based alignment method. The superposed structures for these targets are shown in Fig. 1. This form of visualisation provides, at best, a very broad brush view of features. For example, ligands bind in a well-defined region of the protein, some residues present different degrees of conformational change than others and there are a number of conserved water positions. However it is non-trivial to establish which ligands displace which waters, or move which residues or indeed what pharmacophoric features are most conserved and missing across the available ligands.

## Processing data

The WONKA processing method consists of four steps. First, the PDB [18] files are parsed and the ligands extracted using the RDKit [19] and stored in a Python [20] Django [21] data model. Second, fragments and pharmacophores are generated from the bound ligands and these are again stored in the data model. Third, waters, residues, ligand pharmacophores, ligand fragments and ligands are clustered in space and these clusters stored in the data model. Finally any discovered features are taken from the data model and displayed in an interactive web browser-based application.

### Input of data

The input for each target in WONKA is a single CSV file and the relevant PDB files. The first row of the CSV is the column header. Each subsequent row is a unique ligand-bound protein chain for the target. Models are entered by chain to allow comparison of ligands binding to different chains in the asymmetric unit. If a ligand binds between chains, either chain can be specified. The CSV file should contain a full path to the PDB file for this chain and a SMILES of the ligand bound to this chain, which has assigned the appropriate tautomeric and charge state. Additional information can be provided, including for example the registration ID of this compound and the unique model ID of this chain. An example file is available as part of the WONKA distribution. The PDB files used in this analysis should be pre-aligned to a template chain and the residues should be numbered consistently across all models. In this analysis MolSoft ICM was used, however any protein alignment software can be used. WONKA can then process the data using a simple command line argument.

### Pharmacophore formation and ligand fragmentation

The chemistry of ligands within the available datasets is expressed as pharmacophore and fragment-based abstractions. Ligand-based pharmacophores for all ligands are generated using the standard SMARTS [22] -based RDKit [19] pharmacophore definitions. Halogen and ring-methyl group pharmacophores are also included due to their relevance to medicinal chemistry [23, 24]. A singly substituted non-polar carbon atom is defined as “Hydrophobe” in the analysis below. Ligand-based fragments are generated using the method of Hussain et al. [25] and stored as described previously within our OOMMPPAA method [26].

### Feature clustering

For a given target in WONKA it is not possible to pre-determine the appropriate number of clusters for each feature. However a physically interpretable distance can be used to determine whether two features, e.g. two H-bond acceptors, should or should not fall inside the same cluster. For this reason the Dirichlet process (DP) Means algorithm [27] was chosen. The DP Means algorithm is computationally efficient and does not require a pre-determined number of clusters to be defined. Instead a single parameter (λ) is defined which determines the maximum distance allowed between a point in a cluster and the cluster centre.

The values of λ within WONKA were chosen based upon visual inspection of the three bromodomain datasets in this analysis. They were then validated using several GSK datasets from diverse targets. For waters 1.5 Å was chosen, for pharmacophores and fragments a value of 2.0 Å was chosen, for residues a value of 2.5 Å was chosen and for ligands a value of 5.0 Å was chosen. Clusters are determined and stored in the database for later use. For waters, pharmacophores and molecules the Cartesian coordinates of the unweighted centre of mass are employed for clustering. For residues the all-against-all heavy atom RMSDs between like-residues in the structural ensemble are used. The λ values chosen have been shown to be broadly appropriate for the eight diverse targets analysed by WONKA so far. However this may not be true for all target classes. The parameter can be altered in the source code before analysis takes place.

### Visualisation

The visualisation component of WONKA, which runs within modern web browsers, consists of four main components: (A) the “Key Feature” and “Summary” panels for selecting and displaying important clusters; (B) the 2D compound display and selector; (C) the 3D protein compound display; and (D) the annotation and download tool.

#### Key Feature and Summary Panel

**Fig. 2:**
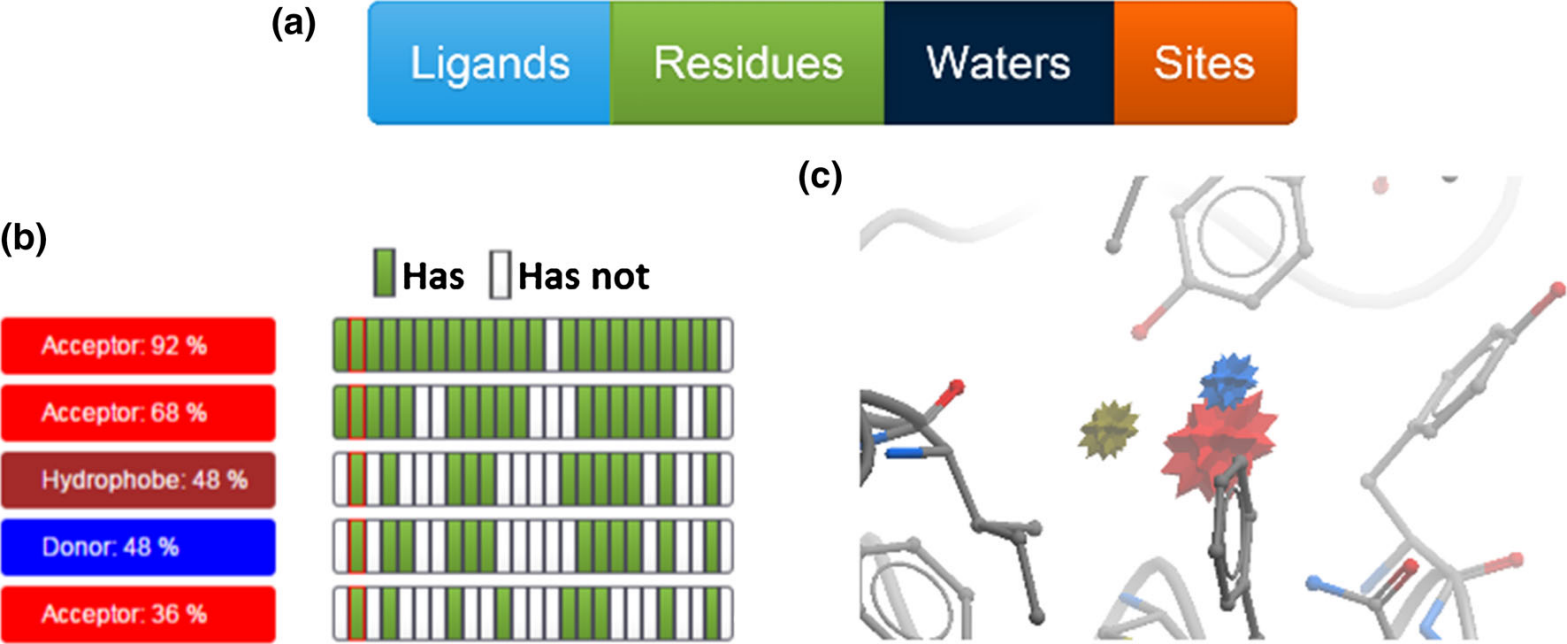
The entry point of the user interface for WONKA. **a** The “Key Feature” panel. Clicking on each button generates a different summary display in section b. **b** The “Summary Panel”, in this case for Key Pharmacophores. Each *row* is a different pharmacophore cluster and each *column* is a different bound ligand. A *green* grid element indicates the ligand contains the corresponding pharmacophore. **c** The 3D display. Ligands can be displayed here by left-clicking on the corresponding column. Pharmacophore features are shown—colour coded corresponding to b

The entry point to WONKA analysis is the “Key Feature” panel (Fig. 2a). The “Ligands” tab in the panel displays the Pharmacophore information in the “Summary Panel”, shown in Fig. 2b. Each Pharmacophore cluster is displayed as a row. They are shown in descending size order, so that the most conserved pharmacophore is highlighted at the top of the list. Clicking on each button displays the cluster centre of mass as a star in the 3D viewer (Fig. 2c). Each star is coloured by the pharmacophore point (e.g. red for H-bond acceptors) and its size scaled according to the percent conservation of this feature (i.e. a larger point is more conserved). In Fig. 2c, the red acceptor feature is more conserved than the blue donor feature or the brown hydrophobe feature. On the right hand side of each row is a grid of green and white buttons. Each column of this grid represents a ligand. If the ligand presents the feature the button is coloured green. If it does not it is coloured white. Clicking on each button displays the ligand in the 3D Viewer and colours the column border red, to indicate this ligand is shown. This assists the user firstly in identifying a consensus pharmacophore for the bound ligands and secondly in isolating ligands that have, or do not have, a given pharmacophore feature.

The “Residues” tab displays all residues within 5.0 Å of any ligand (by default in the most populated ligand cluster) in descending order of maximum RMSD in the “Summary Panel”. For residues the panel on the right hand side is coloured by the cluster this ligand moves this residue into.

**Fig. 3:**
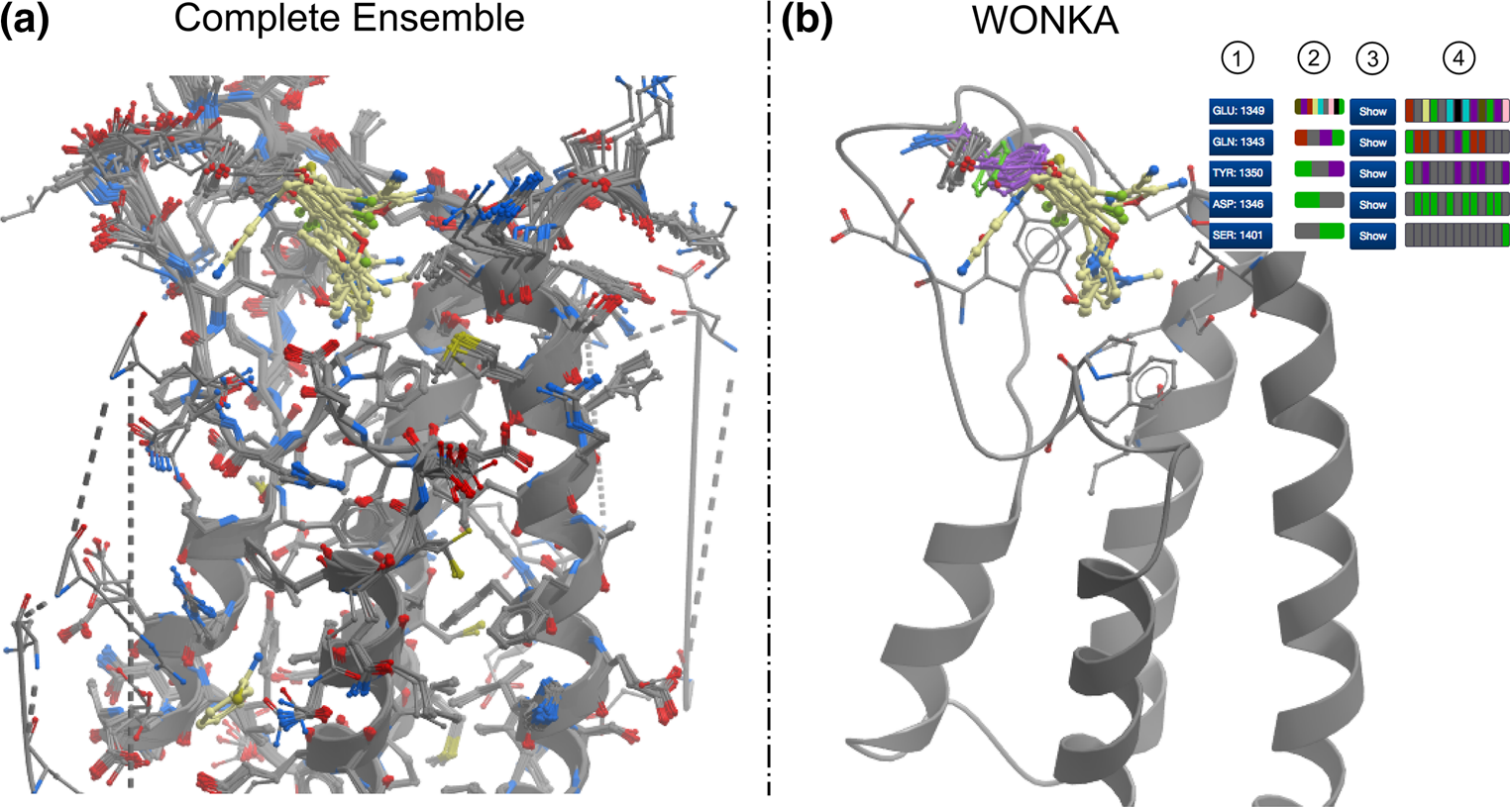
Capturing and presenting interesting residue conformational differences. **a** The complete ensemble of PHIP models is displayed. Some residue conformational differences can be identified but cannot be assigned to specific ligands. **b** WONKA clusters a particular tyrosine’s conformations into *green*, *grey* and *purple* clusters. The residue button (1), highlights each residue. Clicking on the *green*, *grey*, or *purple* buttons, (2) shows only ligands in the *green* cluster, *grey* cluster or *purple* cluster respectively. Clicking on the “Show” button (3) results in only the conformational clusters for this residue being shown. Individual ligands can also be selected, based on their cluster, by clicking the feature summary panel (4)

An example of residue clustering for PHIP is shown in Fig. 3. In Fig. 3a the ensemble of data is shown, from which some changes in residue conformation can be seen. However it is unclear which residues undergo the greatest conformational changes, how many different conformations exist and which ligands are associated to each residue conformational cluster. In Fig. 3b, WONKA highlights a tyrosine residue that presents three distinct conformational clusters (third in list). Clicking on the green, grey or purple button (annotated “2”) shows all the ligand conformations with this tyrosine in the green, grey or purple conformation cluster. Clicking on the relevant “Show” button (annotated “3”) highlights the three conformational clusters for this residue (green and grey stick for carbon atoms) in the 3D display. Equally, by clicking on each grey panel (Fig. 3b annotated “4”) for this tyrosine, the ligands related to the grey conformation cluster are then shown in the 3D display. This example demonstrates how WONKA can efficiently highlight residue conformational differences, cluster them, and then relate them back to the relevant ligands involved.

**Fig. 4:**
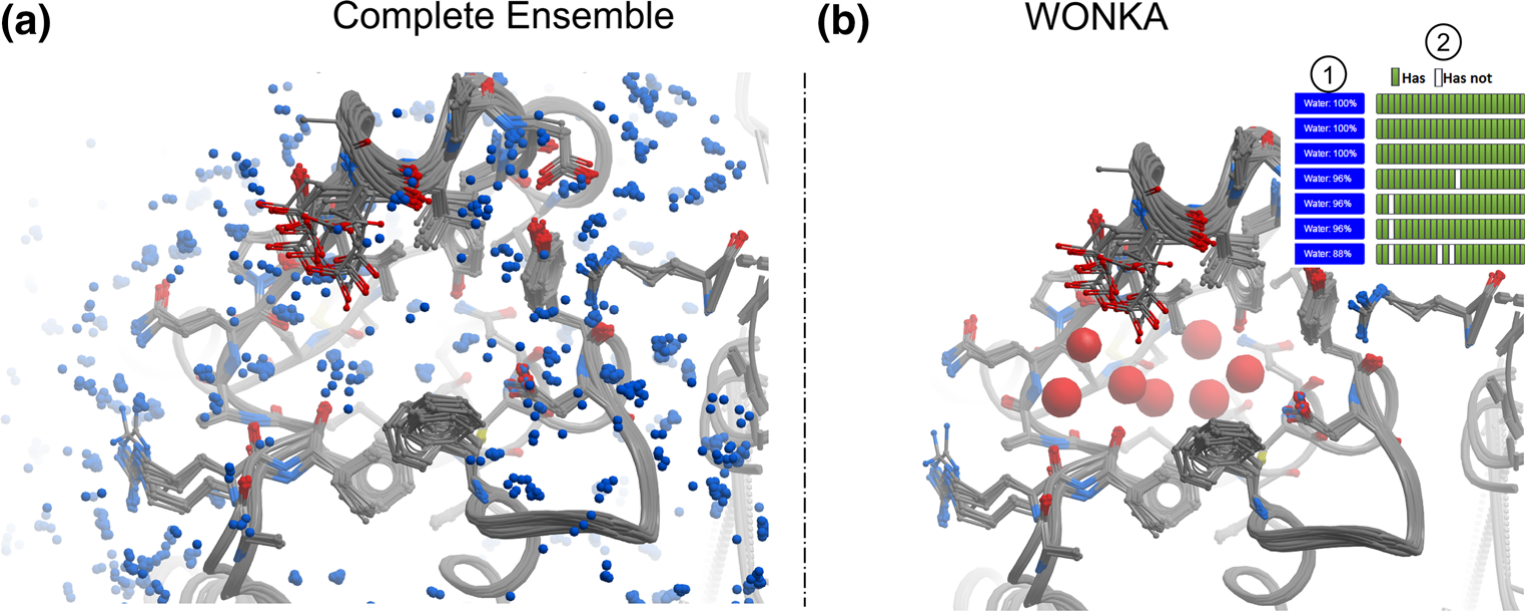
Summarising conserved water locations and highlighting displacements. **a** Displays all the waters for the BRD1 ensemble of structures. Some conservation can be observed, but the extent of conservation and any displacement by ligands cannot be seen. **b** WONKA shows all waters as *red* balls, whose radius is proportional to percentage conservation, that are at locations which are >85 % conserved and within 1.5 Å of the ligands. From the size of the balls the extent of conservation can be seen. From the “Summary Panel” (*inset*), the waters can be shown or hidden (1) and the ligands that displace these waters are highlighted (*white boxes*) and can be displayed (2)

Conserved water positions are also explicitly considered in WONKA. The “Water” button presents water clusters within 1.5 Å of complexed ligands found in the largest ligand cluster. The water clusters are then shown ordered by the number of complexes associated to each cluster, i.e. how conserved each water is. In Fig. 4a the ensemble of water data available for BRD1 is shown, from which water position conservation can be seen, however the extent of conservation and displacement cannot be determined. In Fig. 4b WONKA shows water clusters with >85 % conservation across the ensemble of models. Waters are shown in the viewer with a radius of the fraction of conservation, i.e. a water in a completely conserved location would be represented with a red ball of radius 1 Å. The “Summary Panel” (inset Fig. 4b) indicates the ligands (columns) that displace these waters as white grid boxes. For example ligand three (column three) displaces the fifth, sixth and seventh most conserved waters. In this example WONKA has processed the ensemble information, as shown in Fig. 4a, prioritised information about water position conservation and displacement and then displayed this in an interactive 3D display.

Clicking on the “Sites” button presents ligand clusters. Each cluster centre is presented as a coloured ball, corresponding to the “Summary Panel”. From the “Summary Panel” the user can select all of the ligands binding in a given site on the protein. Furthermore, pressing the “Analyse” button next to the site button presents the WONKA analysis only for the region of this site in a new browser tab. By default, analysis takes place for the main site, so that only that site’s conserved waters and pharmacophores are shown. For many targets allosteric binding can be a powerful tool to modulate protein activity and selectivity—indeed for both BRD1 and PHIP ligands were found in multiple binding sites. Through the “Sites” button the user can rapidly identify these allosteric binders and then move onto the analysis of key residue movements and water displacements, in each region.

The examples in Figs. 3 and 4 demonstrate how complex ensemble data can be interpreted by WONKA via the “Key Feature” panel. Selection of the relevant feature shows filtered, informative data and visualisations in the “Summary Panel”. From these summaries key insights can be more easily found and used for further compound design.

#### 2D and 3D display

The 2D ligand display shows all complexed ligands as 2D depictions. Clicking on each 2D depiction toggles its display on and off in the 3D display. Control-click toggles the display of its complexed protein, shift-click its complexed waters. Activity data can be added as outlined for OOMMPPAA [26] and can be viewed by hovering over the 2D compound depictions. The “Order Chem” button sorts the compounds based upon Morgan fingerprint [28] clustering (radius 2) carried out using the RDKit Butina clustering algorithm [29]. The 3D display is an interactive 3D window for displaying the protein, ligands, waters and key features and is powered by the activeICMJS 3D protein viewer [30].

#### Structure annotation

A unique feature of the interactive portion of WONKA is the “Annotation and Download Tool”. This feature improves upon earlier tools such as iSee [30, 31] and ChromoHub [32], which have been used successfully to annotate and summarise structural data and facilitate the download of integrated datapacks. These tools, however, require significant manual effort in generating observations. WONKA provides an easy-to-use and easy-to-share tool for making such annotations, which reduces the time spent on duplicated analyses of structural ensembles and allows for expert analyses to be shared across the community.

**Fig. 5:**
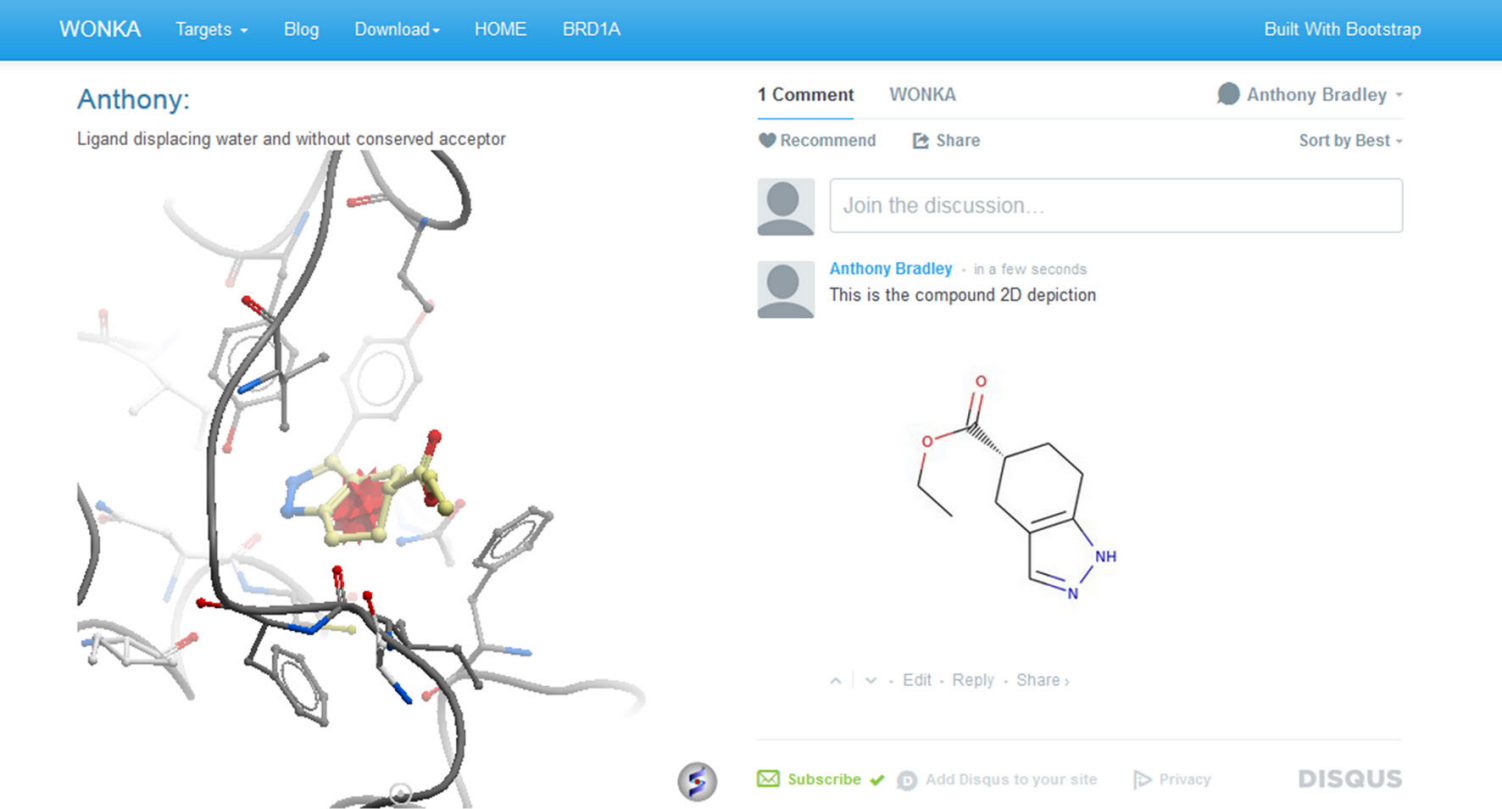
An example WONKA comment. *Left*, the fully interactive activeICMJS panel displaying the observation and comment (PDB code 4AMF). *Right*, the Disqus discussion allows for dialogue on this observation, including image upload and comment prioritisation by moderators

During the analysis of the structural ensemble in WONKA a user can, at any point, capture an observation using the “Save State” button below the 3D viewer, after entering any comments they wish to share. This saves the comment and the view in the back-end data model and creates a unique URL to share this observation. An example of this is shown in Fig. 5 and is accessible here: http://bit.ly/19EVO21.

On the left hand side the Author, the Author’s Comment and a 3D fully-interactive display of the view captured is displayed. On the right hand side a Disqus [33] discussion panel is presented allowing multiple users to comment and discuss the observation in an online forum-like manner. Observations can be filtered and selected for a specific target. In the future, further meta-data will be provided to filter the search on other factors, e.g. the author of the comment, compound or residue shown. This capture, comment and share feature of WONKA reduces duplication of analysis of ligand ensembles and can be used as a training resource, highlighting the important features of protein–ligand interactions for a given target.

## Results and discussion

Below, we highlight examples of how WONKA can be used with three Human bromodomain targets (BRD1, PHIP and BAZ2B), using crystallographic data outlined in the Methods (above). Firstly we demonstrate how water-based analyses can compare and contrast these proteins. Secondly we consider the ligand pharmacophoric differences of PHIP and BRD1. Finally we use WONKA to find and then annotate structures of interest for BRD1.

### Water analysis of three bromodomains

Waters are key components in structure based drug design and their displacement is often important in driving potency and selectivity for many target classes [34, 35]. Bromodomains present an interesting example of water conservation. A wide-ranging structural analysis of many members of the bromodomain family has shown a consistent pattern of four conserved waters at the bottom of a conserved pocket which binds histone peptides [36, 37]. Much work has been done, experimentally and computationally [38, 39], to assess the importance of these waters.

**Fig. 6:**
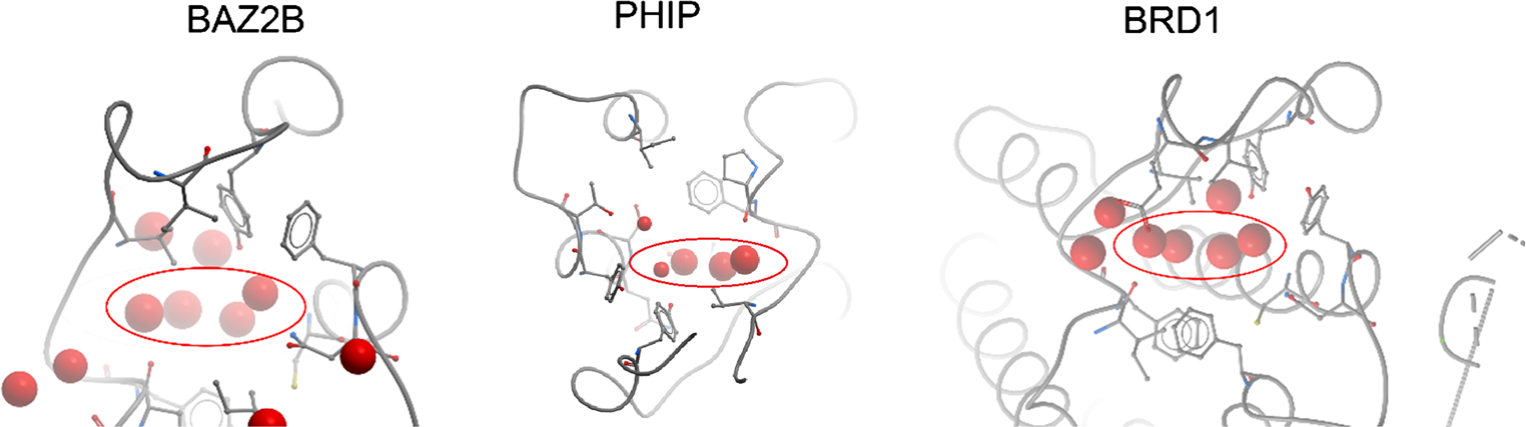
The conserved water positions for BAZ2B, PHIP and BRD1 (*left*-to-*right*) are shown as *red* balls. The radius of the balls is proportional to the fraction of conservation of the water cluster. WONKA highlights the four conserved “bromodomain waters” (*circled*). It also demonstrates the remaining waters follow different patterns for the targets. PHIP’s left most “bromodomain water” is frequently displaced. BAZ2B presents conserved waters outside of the peptide binding pocket. PDB IDs 4CUP, 3MB3 and 4AMF respectively

In Fig. 6 WONKA screenshots are shown of the analysis of the most conserved water-occupied positions for BAZ2B, PHIP and BRD1 respectively. For BAZ2B and BRD1 only waters conserved across 85 % or more structures are shown. For PHIP all conserved waters >50 % are shown. In each case WONKA correctly identifies the four conserved “bromodomain waters” [37] circled. The left most “bromodomain water” for PHIP is smaller than the other three. This indicates that it is less conserved, i.e. more frequently displaced, than the other “bromodomain waters”. Through the “Summary Panel” WONKA can then be used to identify which ligands displace this and other waters, as discussed above.

WONKA also indicates the difference in water locations for these targets. For example BRD1 has two further conserved water positions closely connected to the four bromodomain waters and another further out of the pocket. Conversely BAZ2B has two conserved water positions placed more distantly and a further four out of the pocket. In this example WONKA is able to summarise data from multiple targets. It is able to correctly identify core water structures, indicate which ligands displace these waters and indicate subtle differences in water structures between closely related targets. All of these insights could be leveraged in SBDD programmes to develop more potent and selective molecules.

### PHIP and BRD1 pharmacophore comparison

**Fig. 7:**
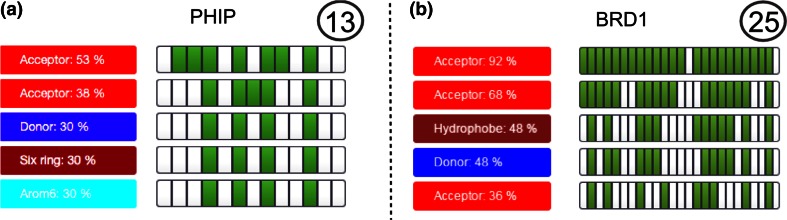
The pharmacophoric conservation comparison between **a** PHIP and **b** BRD1. Firstly the greater number of *columns* in the “Summary Panel” for BRD1 shows that there is less structural data available for PHIP (13 structures) than BRD1 (25 structures). Secondly, whilst BRD1 contains a highly conserved acceptor feature (*first row*)—present in all but two ligands—PHIP does not. WONKA allows the user to scrutinise the ligands missing the conserved features by clicking on the appropriate (*white*) grid box

WONKA can also be used to analyse and compare targets based on ligand-based pharmacophores. Using the “Ligands” button the pharmacophoric conservation of ligands bound to a target can be assessed. The five most conserved pharmacophoric features for PHIP and BRD1 respectively are shown in Fig. 7 from which two observations can be made. Firstly it is immediately clear that less structural data is available for PHIP (13 structures) than BRD1 (25 structures), since more columns are presented for BRD1. Secondly, whilst the BRD1 ligand-set contains a highly conserved acceptor feature—present in all but two ligands—the PHIP ligand set does not contain any highly-conserved pharmacophoric features. The pattern shown for BRD1 is also seen for BAZ2B (data not shown) and is commonly observed across many bromodomains, where an H-bond acceptor is placed forming an interaction with the terminal amide of a conserved asparagine residue [36]. This asparagine residue is not conserved in PHIP, instead it is replaced with a threonine residue. The top-most acceptor cluster in Fig. 7a interacts with this threonine via a water-mediated hydrogen bond, however it is not essential for binding. Investigation of the data indicates that ligands interact with the threonine in a multitude of binding configurations, forming a range of H-bond donor and acceptor interactions, some water mediated. In this example WONKA summarised the key pharmacophores for a set of ligands. Comparison of these pharmacophores highlighted differences between two closely related but crucially different targets. Further, in the case of PHIP, WONKA acts as an entry point for categorising ligands into different interactions with the important threonine residue.

### Finding uniquely bound ligands for BRD1

The “Summary Panel” in Fig. 7b shows that there are only two ligands that lack the most conserved H-bond acceptor for BRD1 (as represented by the two white grid elements in first row). As discussed, this acceptor is a very commonly found feature within bromodomain inhibitors as it forms a critical H-bond with the conserved asparagine residue [40]. WONKA can be used to display these two ligands for further detailed investigation.

**Fig. 8:**
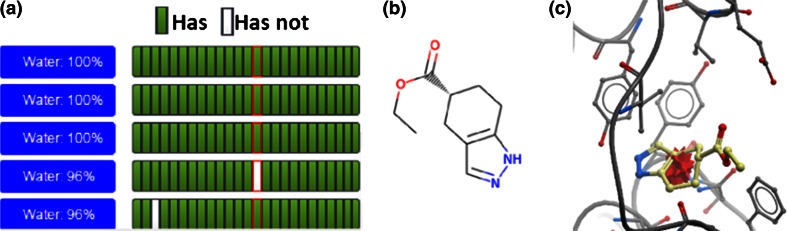
An unusual ligand for BRD1, highlighted in a *red outline* in (**a**). This also shows it lacks the fourth most conserved water and is the only ligand to do so in the data set. **b** The selected ligand in 2D. **c** The 3D view of this ligand’s binding mode (PDB code 4AMF). The red star indicates the position of the most conserved acceptor it lacks. The link to observe this within a WONKA comment is available at the following URL: http://bit.ly/19EVO21

Analysing the ligands through WONKA presents two different stories. By clicking the “Sites” button in the “Key Feature” panel it can be shown that the first ligand binds in an alternative site to the main cluster (data not shown). This ligand would therefore be a candidate for exploring allosteric binding. In contrast, the second ligand binds as expected in the bromodomain peptide binding pocket. However, as shown in Fig. 8c it binds deeper into the pocket, displacing a conserved water molecule and forming a hydrogen bond with a tyrosine residue. In Fig. 8a we show the “Waters” “Summary Panel” for BRD1, with this ligand highlighted in red. Figure 8a shows it has displaced the fourth most conserved water, and is the only ligand in the set to displace it. In this example WONKA quickly indicates two interesting features related to this ligand and BRD1 more generally. The above observations could be of interest to other members of the community. WONKA’s observation capture, annotation and sharing tool was therefore used and the comment is available at the following URL: http://bit.ly/19EVO21.

### Error detection for BRD1

**Fig. 9:**
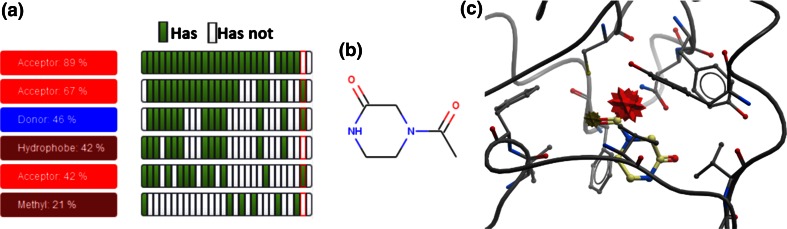
The ligand bound to BRD1 was incorrectly modelled. **a** WONKA shows the *red* highlighted ligand misses three of the six most conserved pharmacophores. **b** The 2D depiction of this ligand. **c** Indicates that rotation of the terminal carbonyl 180° would satisfy the conserved acceptor pharmacophore (PDB code 4AME). A WONKA comment for this is available here: http://bit.ly/1C4BMIM

WONKA can also be used for detecting and reporting possible errors in crystallographic refinement. A previous version of the “Ligands” “Summary Panel” for BRD1 is shown in Fig. 9a. The column outlined in red corresponds to the ligand shown in Fig. 9b and indicates it is missing the acceptor, hydrophobe and ring methyl pharmacophores in the BRD1 ligand set, found in many other ligands in the set. Figure 9c shows these key groups as red (acceptor) and brown (hydrophobe group) stars. The positioning of the features in Fig. 9c indicate that rotation of the terminal carbonyl 180 degrees would satisfy both of these pharmacophore groups. A report of this observation within WONKA can be seen here: http://bit.ly/1C4BMIM. Errors of this nature are easy to miss, particularly for crystallographers unfamiliar with the important binding interactions of a particular target. WONKA makes is easier to identify, highlight and share such issues.

The above examples demonstrate ways in which WONKA can be used quickly and objectively to summarise large and varied ensemble datasets such as those shown in Fig. 1. Key ligand-based, water-based and residue-based features can be observed and then compared between targets. These comparisons may be employed by medicinal chemistry to create potent and selective compounds. Secondly WONKA can be used to quickly find and summarise interesting and erroneous compounds. These observations can then be shared around the community, trivially, using WONKA’s in-built reporting mechanism.

## Conclusion

In this paper we present WONKA, an automated and freely available web-based computational tool to provide analysis of protein–ligand structural ensembles. WONKA aims to consolidate the varied data from an ensemble of liganded structures and provide focussed summaries of this data. These summaries can then be used to describe and compare targets and find erroneous or interesting ligands. This analysis is not trivial using currently available techniques. WONKA also provides a novel data annotation and reporting tool that is, to our knowledge, currently unique within the structural biology community.

WONKA differs from existing tools in three key ways. Firstly it produces combined analyses in a single and intuitive workflow. Secondly it directly relates these analyses to individual ligands allowing for nuanced analysis. Finally WONKA facilitates data annotation and the sharing of these annotations through a unique data capture and storage feature. We have shown varied examples of WONKA’s ability to concisely highlight useful information from large ensembles of structure. From this, evidence-based decisions on future compound design and structural refinement can be made. For these reasons WONKA may serve as an invaluable tool at the initial stages of SBDD and FBDD programmes. WONKA is available to try online or to install locally at http://wonka.sgc.ox.ac.uk/WONKA/.
